# Early Programming of Adult Systemic Essential Hypertension

**DOI:** 10.3390/ijms21041203

**Published:** 2020-02-11

**Authors:** Verónica Guarner-Lans, Abril Ramírez-Higuera, María Esther Rubio-Ruiz, Vicente Castrejón-Téllez, María Elena Soto, Israel Pérez-Torres

**Affiliations:** 1 Department of Physiology, Instituto Nacional de Cardiología “Ignacio Chávez”, Mexico City 14080, Mexico; esther_rubio_ruiz@yahoo.com (M.E.R.-R.); vcastrejn@yahoo.com.mx (V.C.-T.); 2 Nutrition Biochemistry Laboratory, Research and Food Development Unit. Veracruz Technological Institute, National Technological of Mexico, Veracruz 91897, Mexico; abrilhiguera@hotmail.com; 3 Department of Immunology, Instituto Nacional de Cardiología “Ignacio Chávez”, Mexico 14080, Mexico; mesoto50@hotmail.com; 4 Department of Cardiovascular Biomedicine, Instituto Nacional de Cardiología “Ignacio Chávez”, Mexico 14080, Mexico; pertorisr@yahoo.com.mx

**Keywords:** essential systemic hypertension, early programming, epigenetics, endothelial cells, vascular smooth muscle cells

## Abstract

Cardiovascular diseases are being included in the study of developmental origins of health and disease (DOHaD) and essential systemic hypertension has also been added to this field. Epigenetic modifications are one of the main mechanisms leading to early programming of disease. Different environmental factors occurring during critical windows in the early stages of life may leave epigenetic cues, which may be involved in the programming of hypertension when individuals reach adulthood. Such environmental factors include pre-term birth, low weight at birth, altered programming of different organs such as the blood vessels and the kidney, and living in disadvantageous conditions in the programming of hypertension. Mechanisms behind these factors that impact on the programming include undernutrition, oxidative stress, inflammation, emotional stress, and changes in the microbiota. These factors and their underlying causes acting at the vascular level will be discussed in this paper. We also explore the establishment of epigenetic cues that may lead to hypertension at the vascular level such as DNA methylation, histone modifications (methylation and acetylation), and the role of microRNAs in the endothelial cells and blood vessel smooth muscle which participate in hypertension. Since epigenetic changes are reversible, the knowledge of this type of markers could be useful in the field of prevention, diagnosis or epigenetic drugs as a therapeutic approach to hypertension.

## 1. Introduction

The field of study that seeks to explain the way by which the early life environment leaves an increased risk of developing chronic diseases in adulthood, and the mechanisms involved in this programing is known as “Developmental origins of health and disease” (DOHaD) [1]. When the environment in which the individual lives during adulthood is significantly modified from that in which development occurred, the risk of developing diseases increases [2]. Explanations derived from DOHaD may be crucial in the understanding of complex and multifactorial diseases and are now being considered as an alternative to epidemiological explanations [1]. Cardiovascular diseases (CVDs) are being included in the study of DOHaD, particularly coronary artery disease [3,4] and essential systemic hypertension is also nowadays been included. Epigenetic modifications are one of the main mechanisms leading to early programming of disease having an adaptive value since they aim to match the individual’s responses to the environment in which the individual grows, to that in which he will develop during adulthood. Both environments, although supposed to be similar, often differ significantly.

Nongenomic tuning of the phenotype through epigenetic changes such as DNA methylation, histone modifications and non-coding RNAs participate importantly in mediating the impact of the early life environment on later health and its heritability [5]. Epigenetic modifications depend on union and release of chemical residues on a DNA sequence and/or on histones, and therefore, they are reversible and potentially treatable [6]. Thus, although epigenetic inheritance is similar to genetic inheritance of DNA in the capacity to transmit acquired characteristics through generations, epigenetic mechanisms differ in their capacity to be reversible through changes in the environment and by variations in nutritional factors.

Methylation of the DNA structure is involved in epigenetic regulation. The DNA methyltransferases family of enzymes is in charge of catalyzing these reactions. DNA methylation occurs in cytosine-phosphate guanine (CpG) sites. The CpG islands are regions that have an elevated number of CpG sites, and they are often found close to promoter and regulatory regions. DNA methylation of CpG sites in promoter regions inhibits gene expression [7].

Gene expression is also epigenetically determined by structural changes in histones. The histones H2A, H2B, H3, and H4 are organized as an octamer of 4 dimers each that constitute the nucleosome which is the basic structure of organization on which genomic DNA is coiled. H1 histones bind one nucleosome to the next. The amino ends of amino acids conforming histones may be altered by acetylation, methylation, phosphorylation, ubiquitination, and SUMOylation constituting epigenetic cues. Acetylation and methylation of histones are the most common modifications [7]. The consequence of histone acetylation is DNA unwinding and opening, therefore facilitating the access of transcription factors and gene activation [7]. The enzymes that participate in the binding of acetyl groups to histones are histone acetyltransferases (HATs). There are also enzymes that remove acetyl groups from histones known as histone deacetylases (HDAC) and these enzymes have, as a consequence, an inhibition of the access of transcription factors. Gene activation or inactivation may also be elicited by histone methylation having leading to the recruitment of positive and/or negative transcription factors [7].

Non-coding RNAs also play an important role in epigenetic determination. Most of the transcripts of RNA are not translated into proteins and constitute the non-coding RNAs. Non-coding RNAS were originally thought to only regulate gene expression at the post-transcriptional level; however, they have now been found to play an important role in epigenetic control. Non-coding RNAs include microRNSs (miRNAs), Piwi-interacting RNAs (piRNAs), endogenous short interfering RNAs (siRNAs) which have a length of 20 to 30 nucleotides, and long non-coding RNAs (lncRNA), which are more than 200 nucleotides in length. Non-coding RNAs bind to DNA sequences or messenger RNAs inhibiting their transcription or translation or induce their degradation. Long non-coding RNAs are the most common regulatory RNAs [7,8].

Epigenetic changes are importantly established during critical windows of development. A critical window (sensitive period) is a lapse of time during development during which an organism’s phenotype can be modified in response to internal or environmental factors. Windows are phases of developmental phenotypic plasticity and are a consequence of the interaction between the genotype and the environment. Critical windows comprise short periods in development with defined by starting and ending times [2]. Since DOHaD is currently expanding, new windows of susceptibility to modified environmental factors are now being taken into account, such as preconception [9,10], gestation [11], birth, weaning [12] and prepuberty. Moreover, epigenetic alterations in the mother and father are issues in which attention is nowadays being placed when referring to epigenetic programing and transgenerational inheritance [13]. Paternal, in addition to maternal exposure to environmental challenges, are now considered to play a critical role in the offspring’s future health and the transmission of acquired traits through generations [9,10].

Different environmental factors that may leave epigenetic cues are now well known such as pre-term birth [14], low weight at birth [1], altered programming of different organs [15] and living in disadvantageous conditions [16]. Mechanisms behind these factors include: undernutrition, oxidative stress (OS), inflammation, emotional stress, and the microbiota. Many of the epigenetic factors and mechanisms that lead to early programming of coronary artery disease, have been discussed and recently reviewed [3,17,18,19]; however, less has been reported regarding hypertension, particularly at the vascular level. The impact of these factors on hypertension and their underlying causes will be discussed in this paper.

## 2. Hypertension

Systemic hypertension is a common CVD, and is defined by the American College of Cardiology and American Heart Association guidelines as blood pressure >130/80 mm Hg and by the European Society of Cardiology and European Society of Hypertension as >140/90 mm Hg, with the goal of maintaining it at a level <140/90 mm Hg for all subjects and only targeting with treatment to <130/80 mm Hg in patients with high cardiovascular risk [20]. Almost 25% of subjects in the Eastern populations are hypertensive [21]. Although the etiology of hypertension is complex and varies between individuals, a feature commonly found in most cases of hypertension is an elevation in peripheral resistance that is a consequence of increased vascular tone/ smooth muscle cells (SMC) contractility and vascular remodeling [22,23]. These are complex processes that involve phenotypic switching and altered functioning of vascular smooth muscle cells (VSMC). These changes occur in response to modified secretion of vasoconstrictor and vasodilator mediators by endothelial cells. Targets for epigenetic programming of hypertension at the vascular level are enlisted in Table 1 and will be discussed in the following sections. Therefore, both epigenetic changes in endothelial and VSMC may be present in the etiology of hypertension.

## 3. Arterial Morphophysiology and Hypertension

Arterial walls are composed of an internal layer (tunica intima) which is the thinnest layer, formed from a single continuous layer of endothelial cells and is supported by a subendothelial layer of connective tissue and supportive cells. This layer is much thicker in larger vessels such as the aorta. In close connection with the tunica intima there is a thin membrane composed of elastic fibers that run in parallel to the vessel. The tunica media is formed by smooth muscle cells and elastic and connective tissues that are disposed circularly around the vessel. The tunica externa is composed of connective fibers and surrounded by an external elastic lamina whose function is to anchor vessels to surrounding tissues. Large arteries are considered as conductance vessels while small arterioles (30–200 μm) constitute resistance vessels. Both conductance and resistance vessels are involved in the physiopathology of hypertension. Thickness is increased and elasticity is decreased in large arteries from hypertensive subjects. This leads to an elevation of the velocity of the pulse wave and an earlier reflection of the wave, resulting in increased systolic pressure and pulse pressure. Resistance arterioles develop a myogenic tone that protects the capillary bed from high blood pressure and control the local tissue blood flow. In resistance vessels, chronic hypertension causes a reduced lumen diameter provoked by hypertrophy of the vascular wall and exaggerated vasoconstriction [24].

Vasoconstriction and/or relaxation are the result of the balance of vasodilatory and vasoconstrictor substances secreted by the endothelium which act on smooth muscle cells. Endothelial cells constitute a barrier separating the lumen of the vessel and the vascular smooth muscle. They play an important role infiltration of plasma, coagulation and in the secretion of vasoactive mediators. Nitric oxide (NO) is the main vasodilatory mediator. NO deficiency significantly contributes to hypertension [25,26]. Endothelin-1 (ET-1), angiotensin II (Ang II), and other endothelium-derived contracting factors such as cyclooxygenase products may also be released. Endothelium-dependent relaxation is impaired in hypertension and endothelium-dependent contractions may be increased in some blood vessels. These variations in endothelial function result in vasospasm and help to elevate vascular resistance in hypertension.

NO synthesis in endothelial cells occurs through NO synthase (NOS)-independent pathway and NOS-dependent pathway. The NOS-independent pathway involves the reduction of nitrite to NO [27]. This nitrate–nitrite–NO pathway is a complementary pathway to the NOS dependent pathway. There are three isoforms of NOSs, endothelial NOS (eNOS), neuronal NOS (nNOS) and inducible NOS (iNOS) that constitute the main sources of intracellular NO. NO is formed by the reaction that converts L-arginine to L-citrulline; this reaction requires cofactors such as tetrahydrobiopterin (BH_4_), flavin adenine dinucleotide, and flavin mononucleotide. Endogenous NOS inhibitors exist, and can induce NO deficiency including asymmetric dimethylarginine (ADMA) and symmetric dimethylarginine (SDMA) [28]. ADMA competes with L-arginine to decrease NOS activity, having as a consequence a decrease of NO. ADMA also uncouples NOS to generate superoxide [29]. In contrast, SDMA is a competitive inhibitor of L-arginine transport [29].

NO has also been found to have an inhibitory effect on the activity of several sodium transporters [30], and increased expression or activity of sodium transporters and elevated sodium reabsorption might underlie programming of hypertension [31,32]. NO deficiency may not be able to counterbalance the altered sodium transporters induced by early-life insults, thus leading to programmed hypertension.

The renin–angiotensin system (RAS) is a hormone system that regulates blood pressure. When renal blood flow decreases, the juxtaglomerular cells in the kidneys transform a precursor protein normally present in the circulation into renin which enters the circulation. Renin converts angiotensinogen, which is produced by the liver, to angiotensin I. Angiotensin I is then transformed to Ang II by the angiotensin-converting enzyme (ACE) found on the surface of vascular endothelial cells, Ang II is a potent vasoconstrictive peptide that causes blood vessels to contract, resulting in increased blood pressure. When the RAS activity, is too elevated blood pressure will result. The RAS is associated with developmental programming of hypertension in a variety of models, including glucocorticoid administration [33,34,35], high fat diet [36], low protein diet [37], high sucrose diet [38] and high fructose diet [39]. NO inhibition by L-NG-Nitroarginine Methyl Ester (L-NAME) in pregnancy caused programmed hypertension in adult offspring, which was associated with increased messenger RNA (mRNA) of renin and ACE in offspring kidney [40]. Blockade of the classical RAS between 2–4 weeks of age prevented the developmental programming of hypertension [39,41,42,43]. These protective effects were not only directed upon RAS, but also through the regulation of the NO system.

ET-1 is a potent vasoconstrictor. It is a 21 amino acid peptide produced by the vascular endothelium. Several factors increase its synthesis and release such as Ang II, antidiuretic hormone, thrombin, cytokines, reactive oxygen species, and shear stress. ET-1 release is inhibited by prostacyclin and atrial natriuretic peptide as well as by NO [44].

VSMCs are the major components of the tunica media of blood vessels that responds to the vasoactive substances secreted by the endothelium. In adult animals, the VSMC is a specialized cell that regulates blood vessel tone, blood pressure, and blood flow to different regions by determining contraction. Proliferation and synthetic activity of VSMCs in mature blood vessels are very low, and the cells express particular sets of contractile proteins, ion channels, and signaling molecules needed for the cell’s contractile function [45].

Many different factors including alterations in the handling and release of intracellular calcium [46], modifications in membrane potential [47] and the phenotypic expression of the cells contribute to the changes in contractility of differentiated VSMC. During development, and in adult organisms, VSMC show a wide range of phenotypes. The VMSC is capable of major changes in its phenotype in response to changes in its local environment, and therefore, it is not terminally differentiated [45,48]. Contractility is also determined by the degree of differentiation or maturation of the VSMC. Many local environmental cues that determine the pattern of gene expression appropriate for that circumstance are constantly integrated to determine the degree of maturation. There are many different VSMC phenotypes that are needed to perform the functions necessary during development, maturation, vascular remodeling, and disease.

Alterations in vascular structure have been reported with important remodeling of the resistance vessels as a result of changes in VSMC phenotype. These alterations include increased growth, synthesis of matrix materials, reorganization of cell-cell and cell-matrix contacts and apoptosis [23,49].

The change in the phenotype of VSMC is defined as vascular remodeling. This process is characterized by modifications of structure leading to alteration in wall thickness and lumen diameter. Extracellular matrix (ECM) degradation and remodeling indispensable to vascular structure alterations highlight the important role played by metalloproteinases (MMP) in VSMC behaviors. In matured and quiescent vessels, active MMPs are absent or expressed at low levels. But in tissues undergoing abnormal angiogenesis and vascular remodeling, MMPs are markedly expressed, secreted, and activated [50]. MMP-2, MMP-9, MMP-3, MMP-1, and MMP-7 have been recognized in vascular tissue and play pathogenic roles in vascular remodeling via regulating VSMC behaviors [51]. Remodeling can be induced through passive adaptation to chronic changes in hemodynamics and/or through neurohumoral factors including Ang II and ROS. Hemodynamic forces such as shear stress and arterial pressure regulate MMP expression and participate in vascular remodeling. Elevated transmural pressure activates MMP2 and MMP-9 in ex vivo porcine carotid arteries [52]. The progression of hypertension involves two different types of vascular remodeling: inward eutrophic remodeling and hypertrophic remodeling [53].

Abnormal VSMC proliferation, migration, and apoptosis are the main causes of vascular remodeling implicated in multiple vascular disorders, including hypertension, restenosis, and atherosclerotic plaque progression and rupture [51].

## 4. Other Regulatory Systems Participating in Hypertension

### 4.1. Sympathetic Nervous System

The sympathetic nervous system participates importantly in the regulation of arterial blood pressure since blood vessels receive innervations from this branch of the autonomous nervous system. An increased sympathetic nervous system activity is a primary precursor of hypertension. The mechanism that increases the activity in the sympathetic nervous system has yet to be fully elucidated. During the development of hypertension, there are imbalances in several neurotransmitters and neuromodulators, which directly and indirectly contribute to increased release of noradrenaline to the postsynaptic targets of the sympathetic nerves that regulate blood vessels. Among the factors that increase sympathetic nervous activity, the following can be cited: dietary sodium chloride, insulin-glucose excess and nitric oxide deficiency, acute increases in plasma osmolality [54,55].

Furthermore, there are bidirectional interactions among the immune system and the sympathetic nervous system that play a role in the development of hypertension [54]. Inflammation, and in particular adaptive immunity, contributes to the development of hypertension and mice lacking lymphocytes have been reported to be relatively resistant to hypertension caused by Ang II, norepinephrine, or deoxycorticosterone acetate–salt challenge [56]. The central nervous system also orchestrates much of the inflammation caused by Ang II and probably other hypertensive stimuli [56].

Activation of the sympathetic nervous system may contribute to the fetal programming of increased cardiovascular risk. Renal denervation prevents hypertension in male offspring in adulthood in models of fetal programming induced by prenatal exposure to glucocorticoids, placental insufficiency, or maternal diabetes. The mechanism by which renal sympathetic nerve activation mediates the development of hypertension following a developmental insult is not clear but may involve modulation of sodium reabsorption along the renal tubules [57]. Although the sympathetic nervous system participates in blood pressure control, its epigenetic programming will not be addressed in the present review.

### 4.2. The Renal System

The kidney importantly participates in the regulation of arterial blood pressure since it determines extracellular volume. Additionally, renal perfusion pressure regulates arterial circulation and blood pressure. Renal artery perfusion pressure directly regulates sodium excretion and influences the activity of various vasoactive systems such as the RAS system [58]. Hypertension is bidirectionally related to kidney disease. It is a major risk factor for kidney disease and it is the result of kidney disease [15]. Development of the kidney can be altered in response to adverse environments leading to renal programming and increased vulnerability to the development of hypertension and kidney disease in adulthood [15]. NO is a key mediator of renal physiology and blood pressure regulation. NO deficiency is a common mechanism underlying renal programming [15]. Furthermore, the kidney is an organ rich with acetylated lysines, which are found in up to >2000 unique histone and nonhistone proteins, therefore being a target of epigenetic programming [59,60]. Although the renal system participates in the regulation of blood pressure, we do not discuss its epigenetic programming in this review.

### 4.3. The Immune System

The immune system also participates in the initiation and progression of hypertension. During development of this disease, immune cells become activated and enter target organs such as the vasculature and the kidney. T lymphocytes contribute to the pathogenesis of salt-sensitive hypertension. When hypertensive stimuli are present, naive T cells develop into T helper cells such as Th1, Th2, Th17, Treg, and cytotoxic CD8^+^ T cells, depending on the microenviroment surrounding the organs. Hypertensive stimuli activate naive T cells through different mechanisms including neoantigen presentation by dendritic cells, high salt concentration, and OS in the kidney and vasculature. Activated T cell subsets in injured organs promote tissue dysfunction favoring sodium retention in the kidney, vascular stiffness, and remodeling in blood vessels [61].

Mediators released by immune cells, such as ROS, metalloproteinases, cytokines, and antibodies cause damage and mediate dysfunction of the target organs [62]. ROS contribute to immune activation in hypertension [63]. In vessels, these factors favor constriction and remodeling. In the kidney, these mediators elevate activation and expression of sodium transporters, promoting interstitial fibrosis and glomerular injury [62].

## 5. Environmental Factors that May Leave Epigenetic Cues for Hypertension

There is increasing epidemiological evidence that suggests a link between low birth weight, infant and childhood growth, adult body mass index, and maternal weight and nutrition to cardiometabolic risk when reaching adulthood in humans [1]. These environmental factors are listed in Table 2. Most studies have focused on the relation of low weight at birth and coronary artery disease. These studies have been done in different countries and populations [1,64]. Correspondingly, several factors acting during early life have been linked to development of hypertension during adulthood but few reviews analyzing this issue have been published. Among the environmental factors that lead to hypertension preterm birth, low birth weight, gestational hypertension, maternal obesity, short-term breastfeeding, maternal smoking, low vitamin D intake and excessive postnatal weight gain may be included [15]. Living in disadvantaged neighborhoods, which implies mental stress, has also been related to the development of hypertension [16].

Pre-term birth has been associated with cardiovascular and chronic kidney diseases [15]. Structural or functional development is arrested in the kidney and factors including antenatal steroids constitute potential mechanisms that lead to programming and epigenetic alterations in the kidney [14]. A case-control study of >1.6 million infants demonstrated that prematurity and low birth weight are risk factors for congenital anomaly of kidney and urinary tract [65] and both of these factors are associated with low nephron number [66]. Renal alterations are linked to hypertension. Preterm infants may develop low nephron numbers as a result of compromised gestation, intra-uterine growth retardation, inadequacy of postnatal nutrition, and treatment with nephrotoxic medication after birth [66]. A reduced nephron number results in a higher glomerular capillary pressure and glomerular hyperfiltration and thus to renal induced hypertension. A low nephron number is commonly found to underlie the vulnerability to kidney disease and hypertension [66]. Furthermore, NO deficiency in developing kidneys induced by ADMA may alter nephrogenesis [67] and may lead to hypertension.

## 6. Mechanisms behind Factors that Leave Epigenetic Cues

Mechanisms behind factors that leave epigenetic cues are listed in Table 2 and described below.

### 6.1. Undernutrition, Changes in Nutrition and Metabolic Adjustments in Utero and during Early Childhood

The Dutch famine birth cohort study demonstrated that adults exposed to maternal famine developed many disorders, including hypertension and kidney disease when adults [68]. Nutrient-sensing signals play a determinant role in metabolism and development of the fetus [69]. When the diet is imbalanced and when the metabolic status during pregnancy alters nutrient-sensing signals, programming is manifested and hypertension development may result [69,70,71]. Several signaling pathways related to the sensing of nutrients have been described such as the cyclic adenosine monophosphate (AMP)-activated protein kinase (AMPK), the silent information regulator transcript (SIRT), peroxisome proliferator-activated receptors (PPARs), and PPAR coactivator-1α (PGC-1α) [72]. There is a complex interconnection between AMPK and SIRTs, which may be determined by the nutrition in the mother. Nutritional interventions might also regulate PPARs and their target genes [69,73]. Molecules that respond to PPAR target genes [74] include NO synthases which regulate NO production in endothelial cells, superoxide dismutases (SODs) which intervene in the control of OS. Furthermore, eNOS-derived NO is capable of activating PGC-1α via AMPK to regulate mitochondrial biogenesis [75]. Mitochondrial biogenesis has been implied in the establishment of epigenetic cues in cardiometabolic diseases [70,76]. AMPK activators and PPAR modulators have been proposed as reprogramming strategies for programmed hypertension and kidney disease [77,78].

Models of fetal hypertension programming have been done using relatively long-term alterations that included gestation and lactation that also resulted in low birth weight in offspring [79,80,81,82]. The decreased growth, in utero, might alter renal development causing hypertension. Rats fed with a high sucrose diet before and during pregnancy and during lactation and the first days after weaning resulted in an elevated proportion of hypertension in the offspring when they reached adulthood [83]. A high salt diet before and during pregnancy as well as during lactation and the early weaning period also elevated the incidence of hypertension [84].

We recently found that a high sucrose intake during a short critical window near weaning (postnatal days 12 to 28 in rats), where there are important changes in nutrition and a critical window in pancreas development, increases arterial blood pressure when individuals reach adulthood. eNOS and SOD2 expression and total antioxidant capacity in rats that received sucrose during the critical window near weaning were decreased when rats reached adulthood and could underly the increase in blood pressure levels. Blood pressure is also elevated after a long-term ingestion of sucrose (seven months). Fatty acids and arachidonic acid only participate in regulation of blood pressure after a long-term ingestion of sucrose. Finally, it is important to control diet during the early stages of development to reduce the risk of developing hypertension when reaching adulthood [12].

When studying a combined maternal plus post-weaning high-fat diet model, resveratrol, an AMPK activator, prevented induced hypertension and elevated protein levels of SIRT1, AMPK 2α, and PGC-1α in the offspring kidney [78]. Resveratrol also reduced the concentration of ADMA in the kidney and OS damage [78].

### 6.2. Oxidative Stress during Early Life

OS plays an important role in hypertension [85] even before birth. Alterations in oxidative balance are caused by nutrient or oxygen deprivation, placental insufficiency, or exposure to toxic substances and other factors where the placenta plays an important role. OS plays an equally important role in fetal stress as a lifestyle and genetic background, which constitute well-known risk factors of CVD. Specific redox alterations in cardiovascular control organs are also suggesting a role of the mitochondria in low weight at birth [86]. Prenatal insults that lead to renal programming and hypertension associated with OS, include maternal undernutrition [87], maternal diabetes [88], prenatal glucocorticoid administration [33,34,89], preeclampsia [89], and exposure to high-fructose diet [90] and high-fat diet [36] in pregnancy and lactation. Importantly, among these programmed models, the impaired L-arginine–ADMA–NO pathway is closely interrelated to OS in determining the programming process [68].

ROS are produced by all vascular cells and have the potential to participate in the pathogenesis of CVD. Moreover, various other cells are also capable of producing ROS and participate in the development of hypertension, for example, immune cells including neutrophils, monocytes, macrophages, and dendritic cells. Although many enzyme systems generate ROS, some are predominant in pathologic processes such as the NADPH oxidases (NOX), uncoupled eNOS, xanthine oxidase (XO), myeloperoxidase, lipoxygenase, cyclooxygenase, cytochrome P450, heme oxygenase, and the mitochondrial respiratory chain. Some of these systems seem to participate in an important manner in hypertension. ROS increase vascular tone by targeting the regulatory mechanisms in the endothelium and by directly influencing the contractility of VSMC. ROS also participate in vascular remodeling by regulating phenotype modulation, abnormal growth and death of vascular cells, cell migration, and ECM reorganization [52].

OS has a deleterious effect on vascular biology via excessive activation of MMPs which regulate VSMC phenotype from secretory to contractile. ROS formation via the NOX-induced by mechanical stretch enhances MMP-2 mRNA expression and pro-MMP-2 release [91]. MMP-9 secretion and activity in monocytes are enhanced by increased NOX dependent superoxide radical production in the atherosclerotic process [92].

Activation of MMPs by ROS is needed for arteriolar remodeling [93]. NOX play an important role in MMP transcription and activation. MMP-2 transcription is induced by NOX4 by stimulating forkhead family of transcription factors (FoxO) activity [94], while phosphorylation of ERK1/2 which is redox-dependent and subsequent activation of MMP-9 after vascular injury is initiated by NOX1 in neointimal VSMCs [95]. Furthermore, ROS-dependent mechanisms activate a variety of other vascular MMP stimuli and modify MMP functions. Homocysteine contributes to arterial remodeling by activating latent MMP-2 through a mechanism that is oxidative/nitrative dependent [96,97]. ROS production is also associated with the stimulation of MMP-1, MMP-2, and MMP-9 expression in cultured endothelial cells and macrophages by high glucose [98,99,100]. Inflammation and OS have also been related to MMP activation induced by smoking which promotes vascular remodeling [101]. Antioxidant approaches are being used to reduce the upregulation of MMPs and attenuate tissue remodeling during vascular diseases [102,103].

One of the alterations found in animals exposed to various fetal stress factors is vascular NO destruction by superoxide anion. NO destruction has been found in fetuses exposed to nutrient deficiency, hypoxia, excess glucocorticoids, or placental insufficiency [104]. Excess superoxide anion interacts with, reducing NO bioavailability by interacting by it and generates ONOO. ONOO is a powerful oxidant that uncouples eNOS through oxidation of tetra hydro biopterine (BH_4_). Thus, a vicious circle of superoxide anion generation is created [105]. Early-life NO–ROS imbalance is capable of programming adult hypertension and kidney disease [106,107]. Inactivation of NO by OS may contribute to the development of hypertension and kidney disease. OS is mainly caused by an imbalance between the oxidants and antioxidant defense system. OS might reduce NO bioavailability by oxidizing cofactor BH_4_ to uncouple NOS, inhibiting DDAH activity to increase ADMA, and scavenging NO by superoxide to form peroxynitrite [26,108]. Therefore, OS induces both class I and II HDAC overexpression and accumulation in the nucleus via the phosphatidylinositol 3-kinase (PI3K)/Protein Kinase B (PKB/Akt) signaling pathway, thus blocking the anti-inflammatory transcription factors NF-E2-related factor 2 (Nrf2) and myocyte-specific enhancer factor 2 (MEF2). Inhibition of Class I HDAC by siRNA or the class I-specific HDAC inhibitor valproic acid, prevented OS-induced endothelial proliferation both in vivo and in vitro [109].

Interestingly, many of these pathways converge on common signaling pathways, such as the mitogen-activated protein kinase (MAPKs) pathway and the phosphatidylinositol-3-OH kinase PI3K/Akt pathway [110]. The MAPK pathway, in particular, can be activated through integrins, NOX, or ion channels, among others. Ion channels activate the MAPK pathway through protein kinase C (PKC) [111] whereas NOX activates the MAPK pathway through ROS [112].

OS is also an epigenetic modulator since ROS induce epigenetic cues contributing to the pathogenesis of cardiovascular diseases including hypertension. Cardiovascular risk factors determine the levels of ROS, which modify the epigenetic landscape by regulation histone modifications, DNA modifications, the expression of non-coding RNAs and ATP-dependent chromatin remodeling [113].

ROS can directly or indirectly affect DNA methylation at the global or local level leading to modulation of gene expression. As a direct effect, strand breaks produced by ROS can result in local hypermethylation. DNA indirect modifications include cytosine methylation, hydroxymethylation or 8-oxo-2deoxyguanosine (8OG) formation. Reduction of the activity of DNA methyltransferases can lead to global hypomethylation [113]. Histone modifications induced by ROS include methylation, acetylation, ubiquitylation, ADP-ribosylation, SUMOylation and phosphorylation [113].

### 6.3. Gut Microbiota and Programming of Hypertension

The GI microbiota also participates in the programming of chronic diseases, such as obesity, chronic inflammatory diseases, type-2 diabetes, asthma and hypertension [114,115]. Furthermore, the gut microbiota may alter the capacity for nutrient processing and absorption thus limiting growth and therefore participate in the development of hypertension during adulthood [5,116]. There are important differences in the fecal microbiota in spontaneously hypertensive rats and Ang II induced hypertensive rats compared to control normotensive animals [117]. Changes were found in microbial richness and diversity and an increase in the *Firmicutes*/*Bacteroidete* phyla. The administration of minocyclin, an antibiotic from the tetracyclin group, equilibrated the gut microbiota in hypertensive animals also diminishing blood pressure. The incorporation of lactic bacteria or fiber that stimulates their growth as part of the diet also normalized the gut microbiota and diminished blood pressure [118].

The ribosomal RNA from genes16S from feces of hypertensive salt-sensitive and salt-resistant rats have been sequenced [119]. Bacteria from the phylum *Bacteroidetes* were more abundant in salt-sensitive rats. Also, the S24-7 family from phylum *Bacteroidetes* and from the *Veillonellaceae* family from the phylum *Firmicutes*, were higher in the salt-sensitive rats. Systolic arterial pressure from salt-resistant rats was normalized when fecal transplants from salt-sensitive rats were done.

On another hand, short chain fatty acids are important signals generated by the gut microbiota that modulate blood pressure. Microbial end products activate the sympathetic branch of the autonomous nervous system and maintain the influx of lymphocytes to the gut [120]. They may also impact renin secretion thus regulating blood pressure [121]. The tight communication between the enteric nervous system and the central nervous system also suggests a link between the microbiota composition and blood pressure regulation [122].

The fetus was thought to be sterile until a few years ago when the presence of bacteria in the placenta and amniotic fluid was demonstrated. Colonization of the human gastrointestinal tract begins at birth [123,124,125,126]. During vaginal delivery, the baby is exposed to microbes belonging to the mother’s vaginal tract and feces. The bacteria present in the hospital or place of delivery and other the external environments, also contribute to the colonization of the newborn intestine [126,127]. The neonatal gut microbiota, in addition to being influenced by the mode of delivery, is also determined by the type of feeding (breast or formula milk), the mother’s diet, the environment and the use of antibiotics and affects its maturation later in life. The passage from a milk-based diet to solid food is also very important in the maturation of the gut flora [123,124,125]. In humans, the assembly of the commensal ecosystem is determined during the first 3 years of life. The microbiota evolves from childhood/adolescence to adult age, being characterized by less diversity between species [126,128]. In adulthood, the gut microbial ecosystem reaches its maturity, becomes more diverse and stable overtime and more resistant to perturbation (i.e., antibiotic use, dietary changes, stress) [126,129].

A variety of metabolic diseases such as obesity, diabetes, and inflammatory bowel disease have been related to alterations in gut microbiota composition [114,130,131]. Microbiota increases pro-inflammatory cytokines via a Nuclear factor-kappa B (NF-κB) pathway, increases intestinal permeability, and elevates OS. Furthermore, since the diet-related gut microbiota influences the production of cardiovascular risk factors indexes such as trimethylamine oxide (TMAO) [132] and inflammation markers such as lipopolysaccharide [133]. Since TMAO is a metabolite that plays a critical role in the pathogenesis of hypertension and in kidney disease, it is possible that combinations of different foods might reduce the production of these markers and be used for therapeutic aims to decrease the risk of cardiovascular problems [134].

### 6.4. Inflammation Leading to Epigenetic Programming of Diseases

The human immune system is characterized by substantial developmental plasticity, and alterations in its development may underlie the association between the early environment and disease. Studies done in the Philippines and lowland Ecuador have shown that nutritional and microbial exposures in infancy act as important determinants of inflammation and diseases associated with it in adulthood. Low levels of chronic inflammation were found in these populations, despite an elevated burden of infectious disease. Thus, early environments modulate the inflammatory responses to stimuli later in life and determine the association between inflammation and chronic diseases [135].

DNA methylation is important in the regulation of inflammatory genes [4]. Promoter hypomethylation of the Toll-like receptor 2 (TLR2) gene is associated with an increased pro-inflammatory response [136]. Chronic inflammation induces an abnormal DNA methylation of some Polycomb group proteins (PcG) that are present in the mammalian genome and that play a critical role in development and differentiation [137,138]. PcG proteins bind to the regulatory regions of target genes and recruit DNA methyltransferases. There is also a cooperation of histone methylation and DNA methylation as a consequence of severe systemic inflammation [139]. DNA methylation and histone modifications may play an important role in the establishment of the epigenetic marks, particularly in the tumor necrosis factor-α (TNF-α) locus [140].

It has also been reported that histone acetylation activates inflammatory genes, whereas increased HDAC activity results in inflammatory gene repression. Acetylation of histone H3 at the promoters of several cytokines and chemokines after inflammation leads to an enhanced recruitment of NF-κB to these regions [141].

miRNAs have also been found by recent investigations to play an important role in the regulation of inflammation development [142,143]. For example, the toll-like receptor 4 (TLR4)-dependent reprogramming of inflammatory genes is mediated by a differential expression of miRNAs (miR-221, miR-579, and miR-125b).

The inducible Jmjd3 enzyme has been described to erases epigenetic marks on histones. It is a member of the Jumonji family of enzymes that provide a link between inflammation and reprogramming of the epigenome [144]. It has been described that continuous IL-4 treatment leads to activation of Jmjd3. Activated STAT6 also positively regulates Jmjd3 by binding to its promoter. Removal of H3K27 methylation marks by Jmjd3 triggers expression of specific inflammatory genes. Jmjd3 also acts through a H3K27 demethylation-independent mechanism [145].

During vascular remodeling, MMPs also function as inflammatory cytokines [146,147]. The important resources of MMPs in vascular tissue are macrophages and neutrophils. MMP expression is stimulated by inflammatory factors, including TNF-α and interleukins (ILs) [148]. Then, MMPs degrade ECM proteins to facilitate migration and recruitment of cells including those of the inflammatory system. Cell surface receptors and other non-ECM molecules are also modified by MMPs, thus altering adhesion, proliferation, and apoptosis of cells in the vessel wall that participate in the inflammatory process [149]. Therefore, MMPs link inflammation with angiogenesis and constitute inflammatory mediators participating as vascular remodeling factors that result in angiogenesis and in vascular remodeling diseases. MMP expression and activation is induced by factors such as hemodynamic changes, OS, inflammation, hormonal factors, and hypoxia.

### 6.5. Exposure to Chronic Stress

The exposure to environmental conditions, such as stress, during critical periods in early life may cause epigenetic programming modifying the development of pathways in the functioning of neural mediators that lead to stable and long-lasting alterations during adulthood determining risk or resilience to cardiometabolic disorders including hypertension. Children raised in low socioeconomic status households under stressful conditions develop hypertension over time and have elevated vascular reactivity to stress [150,151]. The existence of a cross-talk between the ET-1 receptors and the adrenergic pathway has been described [152]; since ET-1 receptor are localized on sympathetic nerves [153]. Thus, the ET-1 vasoconstrictor pathway and stress-induced hypertension seem to be linked [150,154,155]. Reduced ET-1 receptor expression, particularly ET_A_ favors an increase of the adrenergic-mediated responses. Corticotrophin releasing factor, vasopressin, oxytocin, natriuretic hormones, angiotensin, neuregulins, some purinergic substances and some cytokines contribute to long-term modulation and restructuring of cardiovascular regulation networks. The synthesis, release and receptor expression of these mediators seem to be under epigenetic control since early stages of life [70,156].

## 7. Early Programming of Hypertension

There is a decrease in total methylation in DNA of mononuclear cells from the peripheral blood of hypertensive patients. DNA methylation also regulates the expression of genes related to arterial hypertension at promoter sites. These DNA modifications have been found in important genes in endocrine hypertension such as in the hydroxysteroid (11-beta) dehydrogenase 2 (HSD11B2) gene, the somatic angiotensin-converting enzyme (sACE) gene, the Na^+^/K^+^/2Cl^−^ cotransporter 1 (NKCC1) gene, the angiotensinogen (AGT) gene and the α-adducin (ADD1) gene. The regulation of the expression of genes involved in arterial hypertension by post-translational histone methylation at different histone 3 lysine residues has also been found in genes such as lysine-specific demethylase-1(LSD1), HSD11B2, and epithelial sodium channel subunit α (SCNN1A). Noncoding RNAs have also been found to participate in the programming of hypertension, including several microRNAs that modify genes related to steroidogenesis and the renin–angiotensin–aldosterone pathway [157].

An aberrant profile expression of miRNAs has been linked to a series of diseases, including hypertension and miRNA expression in the circulation shows changes in hypertensive subjects. Among the circulating levels of miRNAs that have been found to be elevated in hypertensive patients, the following can be mentioned: miR-221 and miR-222 [158], miR-92a [159,160], miR-21 and miR-1[161] miR-516b, miR-600, miR-605, miR-623, and let-7e [162] and miR-506-3p [163]. Among the miRNAs that have been reported to be reduced in hypertensive patients, there is miR-143, miR-145, miR-133 [161], miR-18b, miR-30d, miR-296-5p, miR-324-3p, miR-486-5p, miR-518b, miR-1236, and miR-1227 [162]. Correlation of other miRNAs with hypertension in peripheral blood mononuclear cells has also been reported. The miR-126 expression was reported to be reduced in these cells of hypertensive patients and, along with miR-9 [161].

In addition, miRNA, have also been linked to OS; miR-200 family members play a crucial role in OS-dependent endothelial dysfunction, as well as in cardiovascular complications of diabetes and obesity. In addition, different miRNAs, such as miR-210, have been demonstrated to play a key role in mitochondrial metabolism, therefore modulating ROS production and sensitivity [164]. By targeting SIRT1, eNOS, and FOXO1 miR-200 impairs their regulatory circuit and promotes ROS production and endothelial dysfunction [165].

### 7.1. Programming of Endothelial Functioning

There is much evidence that suggests that hypertension may be programmed by the dysregulation of the NO system [106,166,167]. NO depletion during pregnancy caused by NG-nitro-L-arginine-methyl ester (L-NAME, an inhibitor of NOS), elevated OS and resulted in the programming of hypertension when the offspring reached adulthood [41,168]. Maternal NO deficiency results in abnormal functioning of many signaling pathways [169], including the MAPK pathway which participated in redox-sensitive signaling, being involved in the development of hypertension [85].

Histone acetylation plays an important role in endothelial gene expression of eNOS [170,171,172]. A specific histone code is responsible for the expression of eNOS [173]; HDAC1 upregulation in ECs leads to eNOS protein deacetylation resulting in a decrease in basal and ET-1 stimulated NO production. HDAC1 knockdown in endothelial cells has as a consequence an increase of both basal and ET-1 stimulated NO production while eNOS acetylation level remains stable. There is also an acetylation-independent mechanism for regulating eNOS activity, possibly through histone deacetylation [174]. Thus, increasing eNOS expression or activity through HDAC1 inhibition could constitute a method to elevate endothelial cell function [175].

HDAC2 may act as a negative regulator of Arginase 2 expression in endothelial cells. Arginase 2 is a competitive enzyme for eNOS substrate L-arginine thus inducing eNOS uncoupling. HDAC2, binds to the Arginase 2 promoter to decrease its expression, resulting in an increase in the production of NO [176]. These data suggest that HDAC2 plays an important role in the maintenance of EC function and aortic relaxation [175]. There is also flow-dependent regulation of gene expression during vasculogenesis which is determined by modulation of HDACs [177]. Shear stress increases p300-mediated acetylation of the p65 subunit of NF-κB and eth expression of eNOS which depends on p300 acetyltransferase activity [178,179]. Moreover, p300 knockdown in ECs diminishes NF-κB expression and of the expression of transcription factors *Activator protein 1*(AP1) and cAMP response element-binding protein (CREB) [180]. These data imply that p300 is essential for NF-κB expression and nuclear translocation, and that HDAC3 counteracts p300 function through NF-κB deacetylation.

DNA methylation may also play a role in programming since hypoxia may increase eNOS expression through the reduction of repressive H3K27me3. This reduction may be linked to an increase of the histone demethylase Jmjd3 [175]. Furthermore, angiogenesis is also controlled by epigenetic silencing of eNOS promotor through methylation of the lysine residue 27 on histone 3 (H3K27me3) [180]. HDACs regulate cell cycle in endothelial cells when blood flow is altered [109]. Cell migration in angiogenesis is modulated by HDAC7 [181].

Not only the synthesis and release of NO are epigenetically regulated but also NO may act as an epigenetic regulator. Recently, NO has been described to play a role in epigenetics and some of its epigenetic properties are still being discovered [182]. NO has been shown to influence key aspects of epigenetic regulation that include histone posttranslational modifications, DNA methylation, and miRNA levels [183].

NO may diffuse from the cytosol to the nucleus or may be directly produced by the nuclear eNOS. It may be produced both by ligand-activated receptors and environmental cues such as shear stress. These stimuli induce the activation of the PI3K/Akt pathway that results in eNOS phosphorylation. Cytosolic NO induces the translocation of nuclear class II HDACs activation and post-translational modifications including tyrosine nitration and S-nitrosylation of transcription factors. In the nucleus, NO may also post-translationally modify HDAC2 and transcription factors.

During differentiation, these processes may lead to the repression of stem and non-mesodermal genes and to the activation of vascular genes [182]. NO determines miR-200a, -200b, -200c, and -429 expressions, which induce meso-endoderm and precursor vascular marker expression.

NO might also participate in the control of chromosomal structure [182]. It inhibits histone deacetylase complexes by enhancing histone acetylation and promotes a chromatin state that supports gene expression. NO might also regulate other targets of redox molecules such as methyltransferases and demethylases [184].

NO participates in histone post-translational modifications (PTMs). Epigenetic effects of NO are mediated through transcriptional regulation of histone-modifying enzymes and by the ability of NO to modulate the activities and cellular localizations of these enzymes through the formation of iron–nitrosyl complexes and S-nitrosothiols [185]. Some of the NO chemical reactions and metabolic processes including S-nitrosylation of thiols, tyrosine nitration, and cGMP production can be linked to chromatin modification.

NO has also been recognized to mediate the epigenetic changes associated with cell cycle arrest and differentiation. HDACs are intranuclear targets of NO, but, due to its highly diffusible nature, it is likely that many other nuclear factors are directly regulated by NO [186].

The primary mechanism that controls ET-1 levels is the rate of transcription from the ET-1 gene (*edn1*). Tissue-specific *edn1* expression is modulated by DNA methylation. Several segments rich in CpG dinucleotides located in the first intron of *edn1* are subject to methylation and gene silencing. Histone modification patterns also influence *edn1* transcriptional activity particularly methylation of histone H3 lysine 4 residues [187].

On the other hand, the administration of ACE inhibitors or Ang II receptor antagonists in early life can prevent the appearance of the disease during adulthood. The expression of the AT(1b) angiotensin receptor gene in the adrenal gland was found to be upregulated causing increased adrenal Ang II responsiveness. The proximal promoter of the AT(1b) gene is significantly undermethylated, and the gene expression depends on promoter methylation [188].

MiRNA from the wall of the vessels have also been found to be modified in hypertensive patients. The role of miRNAs in endothelial dysfunction and hypertension and the molecular mechanisms proposed for miRNA actions may offer novel diagnostic biomarkers and therapeutic targets for controlling hypertension that is associated with endothelial dysfunction. miR-505 was found to be up-regulated in endothelial cells from these patients [189,190]. MiR-17-3p and miR-31, have also been found to be altered in hypertension and they favor vascular inflammation modulating the expression of VCAM-1, ICAM-1, and E-SEL [191,192]. eNOS uncoupling, which decreases NO production, thus contributing to endothelial dysfunction and decreased vasodilation ability, is associated with vascular inflammation and increased OS, and it has been observed that miR-155 regulates endothelium-dependent vasodilation by reducing the eNOS messenger RNA [193]. Additionally, miR-19a shows anti-proliferative properties in endothelial cells by inhibiting cyclin D1 mRNA [194]. Furthermore miR-19b decreases the apoptosis of endothelial cells in the presence of TNF-α [195]. Let-7g, miR-21, and miR-223 may also regulate apoptosis of endothelial cells [196,197,198].

miRNAs participate in hypertension mediated by RAS. The exact role(s) of miRNAs in RAS-mediated cardiovascular inflammation and remodeling is/are still in the early stage of investigation. However, few miRNAs have been shown to play a role in RAS signaling, particularly miR-155, miR-146a/b, miR-132/122, and miR-483-3p [199]. Some miRNAs are associated with the RAS signaling, such as miR-155, miR-146a/b, miR-132/122 cluster, and miR-483-3p [199,200,201]. MiR-145, miR-27a/b, and miR-483-3p decrease the expression of the ACE [202,203]. Several miRNAs that regulate Ang II mRNA, including miR-483-3p and miR-155, are decreased in hypertension, leading to an increase of the expression of this peptide [203,204]. Furthermore, miR-181a inhibits renin mRNA in a genetically hypertensive mouse strain [205] and miR-181a is linked to renin mRNA [206] and is reduced in hypertensive mice. However, this result was not found in humans since miR-181a expression was elevated in the serum and positively correlated with systolic and diastolic blood pressure, independently of renin levels [207].

### 7.2. Programming of Vascular Smooth Muscle

Chromatin remodeling plays an important role in the determination of the phenotype of VSMC [208]. It allows or denies access of transcription factors to marker genes of specific phenotypes, it recruits the transcription machinery appropriate to those genes, and it determines the lineage of the VSMC. All VSMC marker genes and genes that are important for phenotypic switching depend on one or more CArG boxes that are sequences in the promoter and/or intronic sequences of genes to which transcription factors bind [209,210,211]. A box is a repeating sequence of nucleotides that forms part of a transcription or a regulatory signal. The CArG box [CC(A/T)6GG] DNA sequences play a fundamental role in controlling transcription. These boxes are a target of MADS domain proteins (MADS is the acronym referring to the four founding members of the MADS family of proteins that are **M**CM1 from the budding yeast, **A**GAMOUS from the thale cress *Arabidopsis thaliana*, **D**EFICIENS from the snapdragon *Antirrhinum majus* and **S**RF (serum response factor) from the human *Homo sapiens*) that are transcription factors. CArG boxes serve as a binding site for transcription factors such as the SRF (serum response factor), which is similar to the proto-oncogene C-Fos [49]. The SRF-CArG-dependent pathway is important in the regulation of multiple VSMC marker genes. SRF binds to CArG boxes as a dimer [212]; nevertheless, CArG elements are not enough for SMC-specific gene regulation [213,214]. Moreover, a mutation of non-CArG elements including a TGF-control element (TCE) and E-box elements also participate in the expression of VSMC marker genes in transgenic mice [215,216]. TGF increases the expression of most SMC differentiation marker genes, including smooth muscle actin and heavy myocin chain and calponin in cultured SMCs [215,217]. TGF has also been shown to induce expression of a variety of SMC markers in 10T1/2 fibroblasts [218,219].

Histone acetylation is a key for VSMC gene expression [170,171,220]. HAT p300 participates importantly on the differentiation of VSMC regulating phenotypic switching [171]. In differentiating SMCs, there is a decrease in p300 protein levels and an activation of covalent modifications that may cause the factor to migrate from pathways that lead to growth to pathways implied in differentiation.

The data regarding the exact function of specific HDACs in determining VSMC differentiation are conflicting and vary in different models. Although removal of HDAC suppression seems to be needed to maintain VSMC differentiation, the role of specific HDACs still needs to be determined. However, it now seems clear that HDAC2 and HDAC5, down-regulate SMC marker gene expression and HDACs 3, 7, and 8 participate in the differentiation of VSMC and in the regulation of the contractile function.

The access or blockage of specific HDACs and HMTs to histones associated with VSMC marker regions is important. Certain histone tail modifications that include acetylation of H3K9, H3K14, and H4, and dimethylation of H3K4 and H3K79 (but not methyl-H4K20 and methyl-H3K9, which are markers of several non-SMCs and SMC precursors) favor the differentiated VSMC phenotype, while their absence occurs with dedifferentiation, leading to lower expression of SMC markers. These appear to be long term markers of the VSMC lineage. HDAC7 influences vascular development through an alteration of the ECM [181,221] and MMP.

Several factors (such as KLF4, PRISM, and BRG1/SWI/SNF) act as important suppressors and mediators during differentiation, interacting with the myocardin family. They seem to be altered by phenotype-modifying stimuli while chromatin is being remodeled [222].

The phenotype of VSMCs is also regulated by miR-221 which reduces the contractile profile [223]. MiR-153 is high in VSMCs of spontaneously hypertensive rats and may target potassium voltage-gated channels that control arterial contraction [224]. Under physiological conditions, the miR-143/145 cluster is highly expressed in VSMCs and plays a role in the differentiation of stem/progenitor cells into VSMCs. The underexpression of this cluster in hypertension may influence the contractile phenotype of VSMCs [225,226]. MiR-133 acts as a negative regulator of VSMCs’ proliferation [227], while miR-21 upregulation regulates proliferation and survival of VSMCs.

MiR-130a contributes to the VSMC´s proliferation [228] while miR-365 inhibits it by decreasing cyclin D1 expression. The decrease in miR-365 is mediated by the Ang II signaling [229,230]. MiR-26a promotes abnormal proliferation of VSMC and regulates the transcription factors SMAD-1 and SMAD-4 (Small Mothers Against Decantaplegic), two members of the TGF-β (Transforming Growth Factor β) signaling pathway [231]. miR-34b has been found to be decreased in spontaneously hypertensive rats, promoting the proliferation of VSMCs by elevating the levels of cyclin-dependent kinase 6 (CDK6) [232].

## 8. Reprogramming Strategies for Hypertension

Various reprogramming strategies and treatments have been reported for prevention of programmed hypertension. They include supplementation of NO substrate, ADMA-lowering agents, NO donors, and enhancement of the expression and/or activity of NOS modifying epigenetic cues in promoter regions of genes codifying for enzymes involved in these pathways [3,15]. Interventions during pregnancy, infancy, and childhood that may prevent a higher risk of cardiovascular and metabolic diseases later in life are also beginning to be considered, since they may render potential economic benefits and have public health implications [1]. Furthermore, the capacity of epigenetic cues to be reversible renders the field of DOHaD as an important pharmacological target [157].

## 9. Conclusions

It is currently known that CVDs such as systemic hypertension are regulated by epigenetic mechanisms. In this paper, we explain that the pre-term birth, low weight at birth, undernutrition, gut microbial composition, OS, inflammation, and emotional stress, are the factors that impact on the morpho-physiology of blood vessels that may leave epigenetic cues involved in the early programming of hypertension. Since epigenetic changes are reversible, the knowledge of this type of markers could be useful on the field of prevention, diagnosis, or epigenetic drugs as a therapeutic approach to hypertension.

## Figures and Tables

**Table 1:** Targets for epigenetic programming in essential arterial hypertension at the vascular level.

Tissues and Systems Affected by Epigenetic Programming that Determine Functioning at the Vascular Level in Hypertension
In endothelial cells:
Regulation of genes encoding for vasodilator mediators
NO production; eNOS, ADMA
Regulation of genes encoding for vasoconstrictor mediators
ET1
RAS system
In vascular smooth muscle cells
Regulation of genes encoding for markers of VSMC phenotypes
Regulation of genes encoding for metalloproteinases that regulate the VSMC phenotypes
In the Inflammatory System
Regulation of genes encoding for inflammatory mediators that regulate endothelial cell and VSMC functioning
Regulation of genes encoding for enzymes participating in the synthesis of prostaglandins and prostacyclin
In the antioxidant system
Regulation of the expression of genes encoding for antioxidant enzymes that increase the synthesis of NO and of inflammatory mediators

NO—nitric oxide; eNOS—endothelial nitric oxide synthase; ADMA—Asymmetric dimethylarginine; ET1—endothelin 1; RAS—renin–angiotensin system; VSMC—vascular smooth muscle cells.

**Table 2:** Environmental factors and mechanisms that lead to epigenetic cues.

Environmental Factors Leading to Epigenetic Cues	Mechanisms behind Factors that Lead to Epigenetic Cues
Low birth weight	Undernutrition
Decreased infant and childhood growth	Oxidative stress
Adult body mass index	Inflammation
Maternal weight and nutrition	Gut microbiota
	Exposure to chronic stress

## Abbreviations

8OG	8-oxo-2deoxyguanosine
ACE	angiotensin-converting enzyme
AP1	*Activator protein 1*
ADD1	α-adducin
ADMA	Asymmetric dimethylarginine
AGT	angiotensinogen
AMPK	adenosine monophosphate-activated protein kinase
Ang II	angiotensin II
ATP	adenosine triphosphate
BH_4_	tetra hydro biopterine
cAMP	cyclic adenine monophosphate
CpG	cytosine-phosphate-guanine
CREB	cAMP response element-binding protein
CVD	cardiovascular disease
DNA	desoxiribonucleic acid
DOHaD	developmental origins of health and disease
ECM	extracellular matrix
eNOS	endothelial nitric oxide synthase
ET-1	endothelin 1
FoxO	forkhead family of transcription factors
H1 to H4	histones
HATs	histone acetyltransferases
HDACs	histone deacetylases
L-NAME	L-NG-Nitroarginine Methyl Ester
lncRNA	long non-coding RNAs
MADS	acronym referring to the four founding members of the MADS family of proteins that are **M**CM1 from the budding yeast, **A**GAMOUS from the thale cress *Arabidopsis thaliana*, **D**EFICIENS from the snapdragon *Antirrhinum majus* and **S**RF (serum response factor) from the human *Homo sapiens*
MEF2	myocyte-specific enhancer factor 2
miRNAs	micro ribonucleic acids
MMP	metalloproteinases
mRNA	messenger RNA
NADPH	nicotinamide adenine dinucleotide phosphate
NF-κB	Nuclear factor-kappa B
NKCC1	Na^+^/K^+^/2Cl^−^ cotransporter 1
NO	nitric oxide
NOX	nicotinamide adenine dinucleotide phosphate oxidase
Nrf2	NF-E2-related factor 2
OS	oxidative stress
PcG	Polycomb group proteins
PGC-1α	peroxisome proliferator-activated receptors coactivator-1α
PI3K	phosphatidylinositol 3-kinase
piRNAs	piwi-interacting ribonucleic acids
PKB/Akt	Protein Kinase B
PPARs	peroxisome proliferator-activated receptors
RAS	rennin angiotensin system
RNA	ribonucleic acid
ROS	reactive oxygen species
SDMA	symmetric dimethylarginine
siRNAs	short interfering ribonucleic acids
SIRT	silent information regulator transcript
SMAD	Small Mothers Against Decantaplegic transcription factors
SMC	smooth muscle cells
SOD	superoxide dismutase
SRF	serum response factor
TCE	transforming growth factor control element
TGF	transforming growth factor
Th 1- 17	T helper lymphocytes
TLR	Toll-like receptor
TMAO	trimethylamine oxide
TNF-α	tumor necrosis factor-α
VSMC	vascular smooth muscle cells
XO	xanthine oxidase
